# (Magnetic) Cross-Linked Enzyme Aggregates of Cellulase from *T. reesei*: A Stable and Efficient Biocatalyst

**DOI:** 10.3390/molecules28031305

**Published:** 2023-01-30

**Authors:** Dušica Ifko, Katja Vasić, Željko Knez, Maja Leitgeb

**Affiliations:** 1 Laboratory for Separation Processes and Product Design, Faculty of Chemistry and Chemical Engineering, University of Maribor, Smetanova Ulica 17, SI-2000 Maribor, Slovenia; 2 Laboratory for Applied Electromagnetics, Faculty of Electrical Engineering and Computer Science, Institute of Electrical Power Engineering, University of Maribor, Koroška Cesta 46, SI-2000 Maribor, Slovenia; 3 Faculty of Medicine, University of Maribor, Taborska Ulica 8, SI-2000 Maribor, Slovenia

**Keywords:** immobilized catalysts, biocatalysis, enzymes, cellulase activity, CLEAs, mCLEAs, optimization, characterization, kinetic parameters

## Abstract

Cross-linked enzyme aggregates (CLEAs) represent an effective tool for carrier-free immobilization of enzymes. The present study promotes a successful application of functionalized magnetic nanoparticles (MNPs) for stabilization of cellulase CLEAs. Catalytically active CLEAs and magnetic cross-linked enzyme aggregates (mCLEAs) of cellulase from *Trichoderma reesei* were prepared using glutaraldehyde (GA) as a cross-linking agent and the catalytic activity and stability of the CLEAs/mCLEAs were investigated. The influence of precipitation agents, cross-linker concentration, concentration of enzyme, addition of bovine serum albumin (BSA), and addition of sodium cyanoborohydride (NaBH_3_CN) on expressed activity and immobilization yield of CLEAs/mCLEAs was studied. Particularly, reducing the unsaturated Schiff’s base to form irreversible linkages is important and improved the activity of CLEAs (86%) and mCLEAs (91%). For increased applicability of CLEAs/mCLEAs, we enhanced the activity and stability at mild biochemical process conditions. The reusability after 10 cycles of both CLEAs and mCLEAs was investigated, which retained 72% and 65% of the initial activity, respectively. The thermal stability of CLEAs and mCLEAs in comparison with the non-immobilized enzyme was obtained at 30 °C (145.65% and 188.7%, respectively) and 50 °C (185.1% and 141.4%, respectively). Kinetic parameters were determined for CLEAs and mCLEAs, and the *K*_M_ constant was found at 0.055 ± 0.0102 mM and 0.037 ± 0.0012 mM, respectively. The maximum velocity rate (*V*_max_) was calculated as 1.12 ± 0.0012 µmol/min for CLEA and 1.17 ± 0.0023 µmol/min for mCLEA. Structural characterization was studied using XRD, SEM, and FT-IR. Catalytical properties of immobilized enzyme were improved with the addition of reducent NaBH_3_CN by enhancing the activity of CLEAs and with addition of functionalized aminosilane MNPs by enhancing the activity of mCLEAs.

## 1. Introduction

The multimeric cellulase is a system of several enzymes able to synergistically hydrolyze β-1,4 glycosidic bonds of cellulose and produce glucose and soluble sugars [1,2]. Cellulases are complex mixtures of three major types of enzymes: endoglucanases (EC 3.2.1.4), cellobiohydrolases (EC 3.2.1.91), and cellobiases (EC 3.2.1.21). The cellulolytic complex of *T. reesei* is one of the most commonly investigated and studied fungal enzyme systems, which is known to contain at least one cellobiase, endoglucanase I, II, II, and IV, as well as cellobiohydrolase I and II [3]. Cellulase is industrially a very important class of enzyme in connection with the production of second generation biofuels from lignocellulose as biomass [4]. Cellulases are indispensable to various industries and are widely used for breaking cellulosic rigid structures, which can be important for further conversion to glucose, fructose, and 5-hydroxymethyfurfural (HMF). Additionally, the hydrolysis of biomass to sugars is highly dependent on pretreatment procedures, as well as the stability of cellulosic enzymes in the presence of inhibitory compounds, that act as inhibitors of fermenting microorganisms such as phenolics, which are mostly produced during acid pretreatment of biomass [5]. Chemical [6] and biochemical conversion [7] of cellulose and hemicellulose has been extensively studied. The fact that a multimeric and complex cellulase enzyme can easily dissociate and lose its activity shows the importance of preventing cellulase prior to dissociation by protein engineering, chemical cross-linking, or enzyme immobilization [8]. Moreover, the biomass composition contains a complex nature of cellulose and hemicellulose; therefore, various chemical and biochemical conversions of biomass-derived cellulose and hemicellulose have been reported to develop value-added products and biofuels, such as ethanol, biohydrogen, and others [9,10].

However, enzymes are indeed efficient biocatalysts of nature, which can operate in various physiological environments, and are mostly complex and highly sensitive protein molecules with three-dimensional structures, which are essential for certain biological activities. Nevertheless, when enzymes are exposed to specific conditions, changes in conformation may occur, which causes the loss of some specific enzyme features. Such drawbacks can be overcome by the process of immobilization, since immobilization enhances the operational stability [11]. To accomplish a suitable immobilization protocol, limitations such as activity and stability under certain conditions, selectivity and specificity using substrates, as well as enzyme purity must be addressed. When these limitations are properly addressed, immobilization can become a powerful tool in designing a suitable biocatalyst. Each immobilization protocol requires a suitable support choice with suitable properties. Moreover, active groups of the support must immobilize the enzyme. When these aspects are properly selected, the immobilization protocol can lead to a successful immobilization.

The minimum requirements to define an immobilization process include obtaining data on how the enzyme activity is affected by immobilization and comparing the activity of the free enzyme to the activity of the enzyme under immobilization conditions. Expressed activity as recovery activity is the enzyme activity calculated from the immobilization yield and the activity of the free enzyme or the enzyme reference solution. Therefore, expressed activity reflects the effect of the immobilization process on the activity of the enzyme. Another important aspect of a successful immobilization is to determine the protein content in the supernatant. With this measurement, the immobilization yield is determined. The immobilization yield defines the percentage of the enzyme that is immobilized on the support [12]. Even though various methods are used for the immobilization of enzymes, the well-established techniques for enzyme immobilization are encapsulation or entrapment, physical adsorption, covalent binding, and carrier-free immobilization.

Enzyme stabilization may maintain enzyme activity when applied under stress conditions that can cause inactivation. Mostly, this is closely related to polymer structural stability. However, the enzyme activity is lost before the enzyme fully unfolds. While some enzymes can retain a higher percentage of activity when their structure is altered, other enzymes can lose their activity even after minor structural changes appear in their three-dimensional structure. Moreover, enzyme stabilization may be improved after immobilization in any reaction media. In aqueous media, the structural mobility is reduced without affecting the activity of the enzyme. However, in organic anhydrous media, the structural mobility is reduced due to the lack of water, which can improve the stability of the enzyme in some cases. In different cases, the hydrophobic interaction with the organic phase may inactivate the enzyme.

Extensive research was performed by Fernandez-Lafuente et al. regarding stabilization of enzymes via immobilization [13,14,15,16,17,18,19]. Enzyme immobilization must be designed not only to solve the problem of enzyme recovery and reusability, but also to improve enzymes features, such as stability, activity, specificity, and selectivity. However, only a proper immobilization protocol permits an improved feature. Enzyme stabilization can be achieved by only preventing enzyme exposition to some inactivation causes. When using porous supports, an enzyme’s molecules will be inside the particle, which is similar to what occurs when using cross-linked enzyme aggregates or crystals. This means that the enzyme is not able to interact with external interfaces; however, enzymes that are immobilized on nonporous supports are not protected from negative effects caused by interactions with hydrophobic surfaces. In such a case, coating the immobilized enzyme with hydrophobic polymers is a solution. Immobilization can also protect the enzyme from irreversible inactivation caused by aggregation and can also decrease some inactivation caused by partition of some deleterious compounds away from the enzyme environment. Another step in the inactivation of multimeric enzymes is the enzyme subunit dissociation, which can be induced by heat or organic solvents. Enzyme stability can increase in such cases, when enzyme concentration is increased. When pre-existing supports and dimeric enzymes are used, immobilization of enzyme via both enzymes subunits is relatively simple. However, stabilization of multimeric enzymes with larger oligomerization requires the interaction of all subunits with its flat support surface, which is not possible when tetrahedral enzymes are used [20].

Multi-point or multi-subunit immobilization can improve the rigidity and stability of an enzyme, where the rigidification of the enzyme surface of certain areas can also affect the enzyme activity, selectivity, as well as specificity. The selectivity of an enzyme towards specific cleavage sites is influenced by four factors. Firstly, the charge state of different amino acids in the substrate is specific to two or more amino acids (an example is trypsin). Secondly, temperature changes can cause differences in selectivity, as was confirmed for glutamyl endopeptidase [21], where different peptides were obtained at different temperatures after a similar amount of hydrolysis [22]. Lastly, selectivity can also be affected by substrate accessibility, such as by aggregation or folding state [23]. More on this subject is extensively described in a review by Rodrigues et al. [20].

In other cases, the immobilization protocol can be designed to be coupled with purification in just one step, without sacrificing other enzyme improvements. One general strategy, as suggested by Barbosa et al. [17], is immobilization/purification of proteins via antibody-specific adsorption. Such a method can use monoclonal or polyclonal antibodies, which permit extremely selective protein adsorption. Another strategy is immobilization/purification of enzymes and proteins via specific domains, since there are different peptides and proteins which have a high affinity to different structures. Such a structure can be added to the target protein structure by genetic routing and therefore the affinity property can be transferred to the employed protein. The strategy for coupled immobilization/purification of enzymes and proteins via control of the immobilization process can be performed via interfacial activation on hydrophobic supports or as selective immobilization of large multimeric proteins in standard supports. An example of lipase immobilization is extensively presented in a review by Barbosa et al. [17]. However, coupled immobilization, purification, and multipoint or multisubunit immobilization of enzymes and proteins or domain-tagged enzymes and proteins can be performed via covalent immobilization on heterofunctional supports. Additionally, immobilization/purification are also based on different immobilization rates, where the target enzyme has a much faster immobilization rate than other proteins [17].

Carrier-free immobilization of enzymes as CLEAs and their applications are relevant in industrial biotransformations. The selection of suitable immobilization procedures are of great importance and vital to retaining increased relative activities, due to significant variation in immobilization properties, such as relative activity and immobilization yield (protein binding), which can be achieved through various immobilization methods. Successful immobilization results in higher immobilization yield. Hence, immobilization also improves properties of an enzyme, such as performance in organic solvents, pH tolerance, thermal stability as well as functional stability, which not only results in higher activity of each immobilized enzyme, but also increases the protein structural rigidity and stabilization of multimeric enzymes, which prevents dissociation-related inactivation [24]. In the process of cross-linking, glutaraldehyde (GA) is a bifunctional reagent that is used as a cross-linker. In reaction with proteins, it forms a Schiff’s base between the two carbonyl ends of GA and positively charged amino groups on the surface of proteins, to which the best candidate appears to be an amino group of lysine side chains [25,26]. CLEAs offers a lot of advantages compared with alternative immobilization methods: highly concentrated enzyme activity in the catalyst, improved operational and storage stability, better tolerance of organic solvents, enhanced resistance to autoproteolysis and leaching in aqueous media, possible modulation of catalytic properties, and possibility to co-immobilize two or more enzymes as combi-CLEAs. They are easy to prepare and the costs of carriers are eliminated due to its carrier-free immobilization technique [4,27]. Optimization of parameters [28] such as temperature, pH, concentration, stirring rate, precipitant, additives, and cross-linking agent is still largely empirical [29,30] and must be established for each enzyme anew. Activity of the enzyme is related to its conformation, and it is important to optimize the parameters to achieve the suitable activity and stability of the immobilized enzyme. Little attention has been given to CLEAs prepared from lignocellulosic degradation enzymes, e.g., cellulases, xylanases, and pectinases, which have higher commercial importance in the context of lignocellulosic biomass valorization. The limitations of carrier-free immobilizations lay in the fact that crystallization of the enzyme can be replaced by inexpensive and simple precipitates, which form physical aggregates of certain enzymes, without denaturation. Moreover, CLEAs can significantly improve the stability of soluble enzymes in extreme conditions, such as high temperature, organic solvents, and proteolysis. Additional distinct advantages of CLEAs are high specific catalyst surface area, low purity requirement of the enzyme, high operational recovery, and high operational stability. The rigidity of the tertiary structure is improved after CLEAs immobilization, which is the main cause of its stabilization. The cross-linking therefore prevents dissociation of multimeric enzymes and enzyme denaturation is prevented by the multipoint attachment of the enzyme molecules. However, some disadvantages can occur, such as the difficulty of recovering CLEAs particles as well as mass transfer limitations of macromolecular substrates [1,31,32,33]. CLEAs from enzymes with low levels of lysine can also be obtained by co-aggregation with certain polymers, such as bovine serum albumin. However, such co-aggregation can also lead to the formation of clumps due to low compression resistance, which can cause difficulties in separation of CLEAs. CLEAs are not challenge free, while they are of small size and with reduced rigidity, they can be reduced by a drop in pressure, which can hinder their applicability in packed bed reactors, as suggested in a review by Tan et al. [34]. Such limitations can be addressed by amalgamating with non-compressible supports, which indicates the usefulness of different immobilization methods while improving the formulation of immobilized biocatalysts. During CLEA formation, the enzyme molecules are packed in small volume, which leads to a relatively small pore size, which can cause diffusion limitations with macromolecular substrates, such as polysaccharides and proteins, as suggested by Sheldon [35]. Additionally, centrifugation and washing steps can cause further compression of particles. Furthermore, a review by Fernandez-Lucas shows that CLEAs in anhydrous medium behave like hard particles, while when in aqueous medium, their consistency is very poor, similar to gelatin [36]. As suggested in a review by Sampaio et al. [14], another possibility to increase CELAs stability is to use a feeder that is rich in amino groups, such as a protein or an aminated polymer [37,38,39]. Such a solution is efficient; however, it means the feeder increases the cost-related aspects and therefore decreases the advantages of CLEAs in comparison with other immobilization methods. To solve such problems, several reports suggest trapping the CLEAs in more robust particles, which leads to macromolecular magnetically assisted CLEAs, which are easily handled. However, as CLEAs have many advantages, the lack of mechanical robustness still remains an issue. The particle size can be increased by modifying the cross-linking process, while compromising between the ease of preparation of large particles and the lower mass transfer limitations, which decreases activity. In order to overcome such shortcomings, magnetically recoverable CLEAs, known as magnetic CLEAs (mCLEAs), were introduced, where cross-linking is performed in the presence of magnetic nanoparticles (MNPs). As they have small particle size with high activity, they also have the advantage of easy separation on an industrial scale, using magnetic separation with the external magnetic field [35]. mCLEAs are non-toxic, have large surface area with high enzyme loading, and most importantly display magnetic properties, which enable easy recovery of the CLEAs, when the magnetic field is applied thereby eliminating the use of filters and centrifugal assistance [40]. Since the separation of the immobilized biocatalyst from the enzymatic reaction mixture is of key importance, MNPs are the best tool to provide suitable separation with the use of an external magnetic field [41]. MNPs are used as promising carriers in enzyme immobilization technology due to their large surface-to-volume ratio, which provides easy functionalization and stronger binding capacities. The use of MNPs in reaction media can enable facile separation and therefore termination of enzymatic reactions, as well as recovery of the enzymes, which can later be reused. However, when using MNPs in mCLEA preparation, leaching of iron can occur at acidic pH and can be accelerated in the presence of free carboxylic acids. Another limitation is low saturation magnetizability of magnetite, which makes magnetic recovery challenging in large-scale processes. Research by Sheldon et al. overcame such limitations by developing mCLEAs based on non-functionalized MNPs of zerovalent iron [35].

Co-immobilization interest increased with cascade reactions, which is defined as the process where enzyme 1 (E1) is the substrate for enzyme 2 (E2), which involves many consecutively acting enzymes in a more or less complex synthesis route. As suggested in a review by Rodrigues et al., it has been shown that the initial reaction rate can be accelerated when using co-immobilized enzymes. Enzyme co-immobilization also allows access to some kinetic advantages, which are necessary to obtain the desired product while avoiding additional reactions. The kinetic effect is relevant mainly due to the initial reaction rates. However, it can be less relevant to the whole reaction, depending on the kinetic parameters of the involved enzymes. The initial reaction rate is accelerated by co-immobilization due to its reduction in, or even elimination of, the lag time using several enzymes immobilized on different enzyme particles of free enzymes [42]. Many papers have discussed such aspects [34,42,43].

The interest in applying cellulases in the paper and pulp industries has increased during the last decade. For example, grinding of woody raw materials leads to pulps with high contents of fines, bulk, and stiffness. Biomechanical pulping using cellulases can result in substantial energy savings (20–40%) [44]. However, current mechanisms for hydrolysis of lignocellulosic materials are quite expensive and the enzyme cellulose is very sensitive to changes in different environmental conditions. Therefore, using free cellulase in industrial processes is impractical in use because of its low stability and low reusability [45,46]. To overcome these difficulties and to improve practical uses of cellulase in food, feed, and agricultural industry, binding cellulase to solid supports improves cellulase properties, such as its stability, activity, and reusability, making them more approachable to use in large-scale applications. When using solid supports, immobilization to magnetic supports is very desirable due to its ability to simply being able to separate them using an external magnet [47,48,49]. Another advantage in immobilization of cellulase is carrier-free immobilization in the form of cross-linker cellulase aggregates, which provides a more stable structure with a strongly bound enzyme and is a simple and cheap method with high volumetric productivity of the immobilization process [50,51]. Both techniques for immobilization of cellulase are covered in our research. There are some reports on immobilization of cellulase onto mesoporous silica nanoparticles used for cellulose-to-glucose conversion, where 80% yield and excellent stability was achieved [52]. Another study reports on biocatalyst retaining 83% of the initial yield of the reaction after 9 cycles of reuse, which also had better stability than the free enzyme in a wide range of temperatures, preserving 72% of the initial yield of the reaction up to 90 °C [53]. Another article reports of a novel approach for deconstruction of cellulose by integrating a sequential enzyme cascade technique [54]. Immobilization of cellulase enzyme on superparamagnetic nanoparticles via physical adsorption (ionic bounds) was also reported. Stability and activity of the cellulase were enhanced [27]. Cellulase was also immobilized onto MNPs functionalized with triethoxy(3-isocyanatopropyl)silane and iminodiacetic acid, where it was found that the immobilized free cellulase kept 50% of its activity after 2 h, while the activity of immobilized cellulase was observed to be 77% at 60 °C [55]. Another study by Desai et al. reports on iron-tolerant bacterium synthesis of MNPs for cellulase immobilization, which were found to be an excellent support for cellulase immobilization with 96.5% binding yield and 80% retained activity after 3 cycles of reuse [56]. MNPs were also prepared by rapid combustion process, with silica being precipitated on its surface, where immobilized cellulase maintained 71% of its initial activity after 5 cycles of reuse [57]. Magnetic combi-CLEA was prepared using amino-functionalized MNPs and proved to be efficiently reused after 12 cycles, where cellulase retained 88.62% of its activity [58]. Li et al. investigated CLEAs of cellulase using GA as a cross-linking agent, where it showed 65.2% of activity after 10 cycles and 63% of activity after storage for 56 days at 4 °C [59].

The present research paper describes preparation of *T. reesei* cellulase CLEAs and mCLEAs. Catalytically active CLEAs and mCLEAs of cellulose with more than 80% of activity were prepared. Furthermore, aminosilanized magnetic nanoparticles (AMN-MNPs) were used to improve cellulase CLEAs, indicating improved attachment and enzyme conformation. Separation of mCLEAs from reaction mixture is simple, using only magnetic decantation, which eliminates the need for filters and centrifugal techniques [1,2]. Maghemite MNPs were prepared and functionalized with aminosilane, which proved to be useful for the immobilization of cellulase. A novel approach to determine the impact of MNPs on enzyme immobilization is described, where cellulase was immobilized in parallel as catalytically active CLEAs and mCLEAs. Cellulase in a kind of more stable immobilized form may be useful in industrial applications, such as paper and pulp treatment processes, in the food processing industry, e.g., when used as part of a macerating enzymes complex or in the animal feed industry. Furthermore, it can be used for pretreatment of agricultural silage and grain feed, agricultural industries, e.g., for the improvement of the soil quality or for degradation of the cell wall of plant pathogens in controlling plant disease. There are many research opportunities for producing bioethanol from cellulosic materials, which include enzymatic hydrolysis of cellulose or hemicellulose [60,61]. Maximum activity and stability retention of immobilized enzyme was the principal concern. A comparison and evaluation of activity, binding yield, as well as stability of both CLEAs and mCLEAs was studied. The major focus was given to enhancing the applicability of CLEAs and mCLEAs by improving stability and activity of the immobilized enzyme at mild biochemical process conditions by investigating the influence of process parameters, such as selection of precipitation reagents, cross-linker concentration, enzyme concentration, reducing reagent concentration, and by enabling simple separation of the immobilized enzyme from the reaction mixture with the use of AMN-MNPs in the synthesis of mCLEA, which is a novelty in our research.

## 2. Results and Discussions

Varying and studying different reaction parameters in order to select the most suitable conditions to obtain the highest expressed activity and immobilization yield of T. reesei cellulase CLEAs and mCLEAs was required. Different precipitation reagents, various GA concentrations, addition of protein feeder [39], and different concentrations of NaHB3CN were investigated. MNPs were prepared with co-precipitation of Fe^2+^ and Fe^3+^ ions in a molar ratio 2:1 at pH 10 in order to obtain mCLEA. MNPs were characterized in our previous work [62,63]. The optimization of CLEAs and mCLEAs preparation conditions was performed to improve expressed activity of the immobilized enzyme. The entire optimization process was carried out parallelly for both CLEAs and mCLEAs.

### 2.1. Selection of Precipitation Reagent

**Figure 1:**
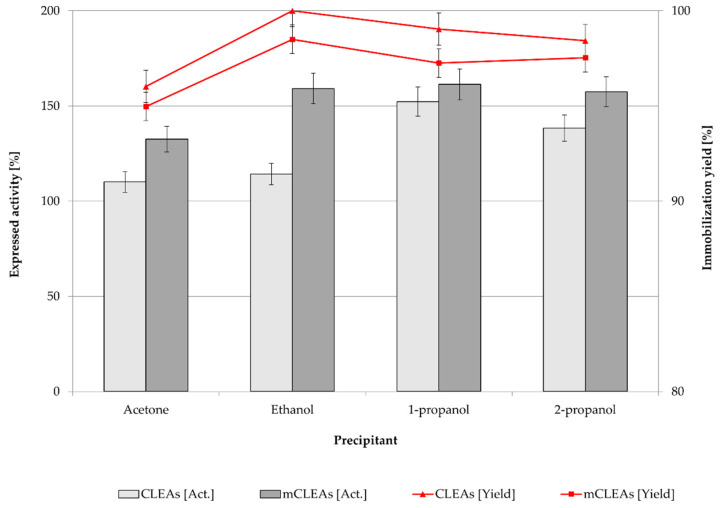
Effect of different precipitation reagents on enzyme activity and immobilization yield of *T. reesei* cellulase CLEAs and mCLEAs (100% of expressed activity equals initial cellulase activity of 9.89 µmol/min mL).

Precipitation is the first step of CLEAs and mCLEAs preparation. Precipitating reagents convert soluble enzymes into insoluble aggregates, which occur by changing their hydration state without affecting the functional properties of the enzyme. As a result, the enzyme expressed activity is dependent on the concentration and the type of the precipitant. Different organic solvents were investigated to determine the most suitable precipitation reagent for preparation of T. reesei cellulase CLEAs and mCLEAs. Four typical precipitation reagents, such as acetone, ethanol, 1-propanol, and 2-propanol were used for preparing cellulase CLEAs and mCLEAs. Cross-linker GA with an initial lower concentration of 0.125 % (*w*/*w*) was studied. The results are presented in Figure 1.

The results in Figure 1 show that cellulase hyperactivation appeared for CLEAs (152.34%) and for mCLEAs (161.33%) using 0.125% (*w*/*w*) GA and using 1-propanol as precipitating reagent. Hyperactivation of an enzyme occurs when the enzyme is highly unstable and the immobilization itself drastically improves the catalytic performance and stability of immobilized enzyme. Most likely this happens due to the conformational changes that occur in the protein as a result of aggregates formation [64]. Immobilization yields were between 99% and 100% for all four organic solvents as precipitation reagents, where ethanol gave the highest immobilization yield, which was 100% for CLEAs and 99% for mCLEAs. Using 1-propanol as a precipitating reagent, immobilization yields were somewhat lower (for CLEAs and for mCLEAs), but still high enough to obtain the highest expressed activity of the enzyme. Our results are in agreement with reports by Peirce et al. [65] and Schoevaart et al. [30]; if the enzyme solution is introduced to a large volume of concentrated precipitating reagent, sufficient results are obtained in terms of precipitation yield and activity of the enzyme aggregates.

In fact, in all four cases cellulase hyperactivation was observed. However, expressed activity and immobilization yield of CLEAs and mCLEAs were expected to decrease after purification with buffer solution (washings); therefore, further stages of optimization were performed.

### 2.2. Effect of Cross-Linker (GA) Concentration

**Figure 2:**
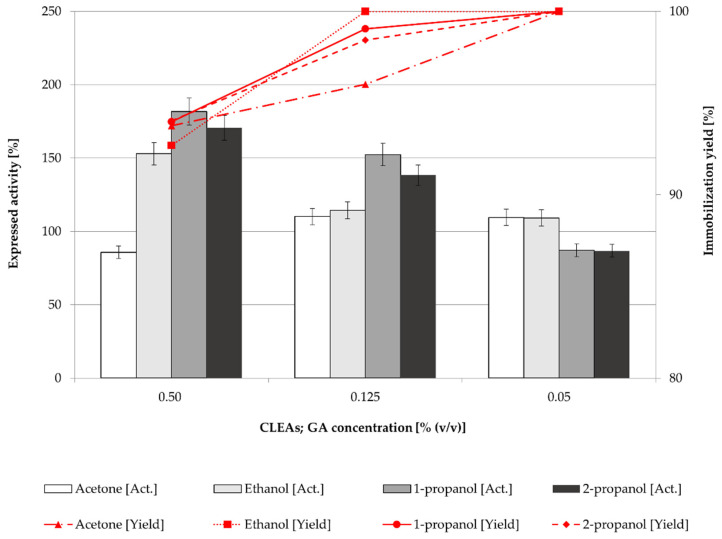
Effect of GA concentration on the CLEAs activity and immobilization yield (100% of expressed activity equals initial cellulase activity of 9.89 µmol/min mL).

In general, the expressed activity of CLEAs or mCLEAs appeared to be highly dependant on the cross-linker concentration. Concentration of cross-linker is of key importance in CLEAs and mCLEAs preparation, as it influences the activity and stability of CLEAs and mCLEAs, as well as particle sizes. Different concentrations (0.50% (*w*/*w*), 0.125% (*w*/*w*), and 0.05% (*w*/*w*)) were applied and the expressed activity of CLEAs and mCLEAs as well as immobilization yield were determined. In Figure 2, the results suggest that the best precipitation reagent for CLEAs when using GA as a cross-linker in concentrations of 0.50% (*w*/*w*) and 0.125% (*w*/*w*) was 1-propanol, which gave 180.71% (17.87 µmol/min mL) of cellulase CLEAs expressed activity when using 0.50% (*w*/*w*) of GA. This is even higher than that observed in the previous experiment (Figure 1), in which 151.34% (14.97 µmol/min mL) of expressed activity was reported when using 0.125% (*w*/*w*) of GA.

**Figure 3:**
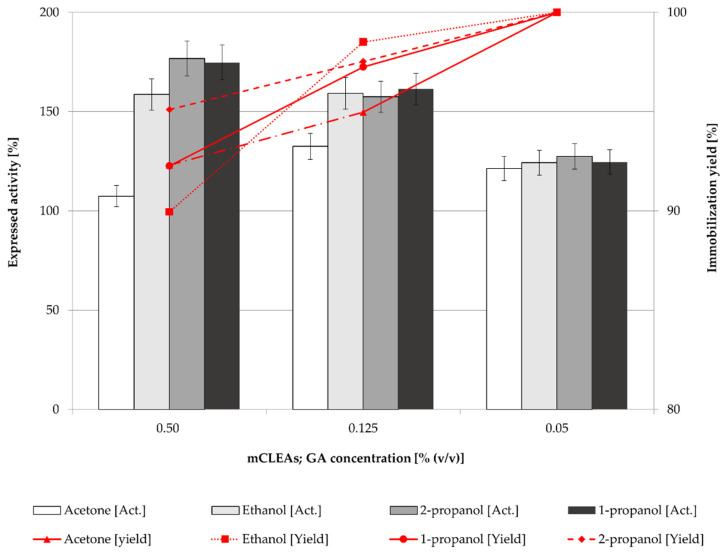
Effect of GA concentration on the mCLEAs expressed activity and immobilization yield (100% of expressed activity equals initial cellulase activity of 9.89 µmol/min mL).

The highest GA concentration also gave the highest CLEAs activity even though the immobilization yield was the lowest. The same trend could be observed for mCLEAs preparation (Figure 3). The differences in immobilization yield are not significant, ranging from 90% to 100%; however, there are significant differences in expressed activities. At lower cross-linker concentrations, insufficient cross-linking occurred, which resulted in unstable CLEAs and mCLEAs releasing the enzyme into the reaction media.

Figure 3 shows that the activities of mCLEAs were higher at higher GA concentrations. Compared with the activity of CLEAs, they were higher when lower GA concentrations were used due to the positive impact of MNP on the cross-linking. They were also more similar for different precipitation reagents at 0.05% (*w*/*w*) and 0.125% (*w*/*w*) of GA concentrations as the activities of CLEAs at the same concentrations of cross-linking agent. However, at a GA concentration of 0.50% (*w*/*w*), activities of CLEAs and mCLEAs using different precipitation reagents were not significantly different, since the impact of MNPs was not apparent. Nevertheless, very high GA concentration may cause excessive cross-linking and loss of enzyme activity [66]. This could also be explained by lower accessibility of active sites of cellulase mCLEAs. Excess GA may also result in intramolecular cross-linking due to its smaller size, which can reduce the activity of an immobilized enzyme. To overcome such limitations, protective additives, such as BSA are used during the aggregation and cross-linking process, often to overcome the low activity and recovery, as well as to protect the structure of the enzyme [67,68,69]. This approach was also performed in the next stage of our study.

**Figure 4:**
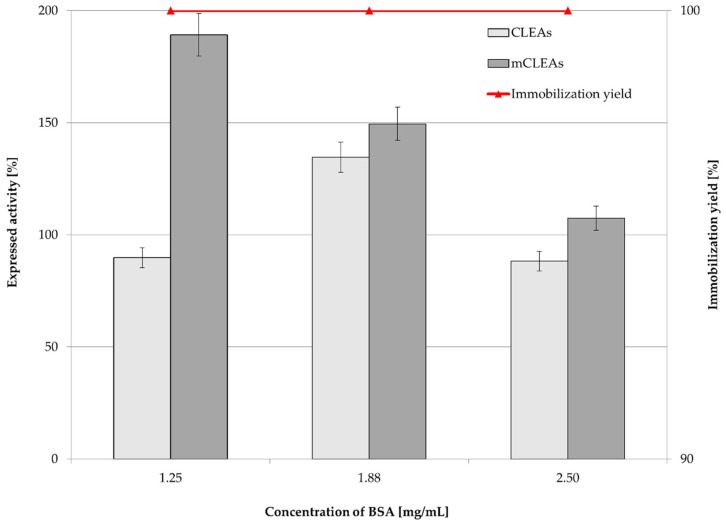
Influence of BSA concentration on CLEAs and mCLEAs expressed activity and immobilization yield (100% expressed activity equals initial activity 9.89 µmol/min mL).

As reported in many articles, in too low concentrations of GA the enzyme is not cross-linked properly, which also means that a low degree of cross-linking causes enzyme leaching from CLEAs or mCLEAs. Such was observed in a study by Nguyen et al. [70] and many others [71,72]. Jung et al. [73] observed that when the GA concentration was too low, the cross-linking was not properly formed. However, when the GA concentration was increased the conformational stability of CLEAs was increased as well. Amadi et al. reported 84% activity recovery of cellulase when applying 50 mM GA into the CLEAs synthesis [71]. Similarly, a study by Lucena et al., where cellulase mCLEAs were synthesized and compared with cellulase CLEAs activity, resulted in 33% higher activity for mCLEAs. Both exhibited high enzyme loading, resulting in 88% and 90%, respectively [74]. Figure 1, Figure 2, Figure 3 and Figure 4 show that mCLEAs show higher expressed activities than CLEAs under the same immobilization conditions. The immobilization yield of CLEAs and mCLEAs resulted above 99% (Figure 2 and Figure 3). The immobilization yield of CLEAs and mCLEAs was the lowest at 0.50% (*w*/*w*) GA, where expressed activities were the highest. However, both expressed activity and immobilization yield were relatively high at 0.125% (*w*/*w*) GA and hyperactivation was present, as listed in Table 1.

### 2.3. Effect of Proteic Feeder and Enzyme Concentration

In the CLEAs synthesis, BSA may be used as a proteic additive, which acts as a stabilizing protein in order to increase the number of lysine residues, therefore providing GA with enough amine groups for cross-linking. In that manner, with an appropriate concentration of proteic feeder that stabilizes the enzyme and with an appropriate concentration of cross-linking agent, the enzyme structure remains undistorted, which allows the active sites of the enzyme to be available for substrate binding. The availability of present amine groups forms a stronger covalent bond, which enhances enzyme recovery. Perwez et al. used a BSA concentration of 0.5 mg/mL, which showed maximum activity recovery. Additionally, GA was used as the cross-linking agent. As it is a bifunctional cross-linking agent, it links the amine group of the enzyme with the aldehydic group, which forms the Schiff’s base. The combi-CLEA with pectinase, xylanase, and cellulase retained 86%, 90%, and 88% activity, respectively [58]. The addition of proteic feeder BSA (at concentrations 1.25 mg/mL, 1.88 mg/mL, and 2.50 mg/mL) was used as it can potentially improve CLEAs and mCLEAs activity and more over its stability, as confirmed by our previous studies [75]. BSA can be used as protein matrix for improving enzyme stability and is usually used while working with low protein concentrations in enzyme preparation. It is possible to assume that it prevents the loss of enzyme activity during the process of precipitation and cross-linking [76]. The concentration of 1.88 mg/mL BSA in the formation of CLEAs and mCLEAs resulted in a slight increase in CLEAs activity, while a decrease in mCLEAs activity was observed. It is possible that amino groups of BSA could get attached to the catalytic domain of the enzyme and therefore decrease its catalytic ability. The immobilization yield was 100% for CLEAs and mCLEAs (Figure 4).

The benefit of using proteic feeder BSA was also reported in a study by Shah et al. [39] and Cruz et al. [37], which is in accordance with our results. The use of proteic feeder is necessary to obtain actual cross-linking of enzyme molecules, while having low density of lysine in its surface. Therefore, BSA may be a good choice as a proteic feeder, as it is a molecule with amino groups on its surface and allows easy cross-linking using GA. Hence, having BSA in the synthesis of CLEAs and mCLEAs leads to extensive cross-linking with high hydrolytic activity. The increase in activity in CLEAs and mCLEAs formation was also observed in previous works, where Galvis et al. reported on increased activities while using BSA in CLEAs [77]. The differences in expressed activities can be attributed to different sizes of particles as well as in different sizes of internal pores, which can modify the diffusion of substrates.

**Figure 5:**
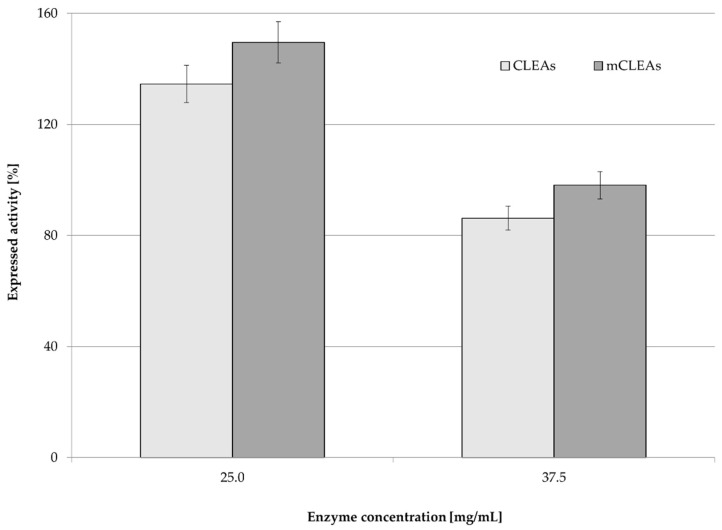
Effect of cellulase concentration on CLEAs and mCLEAs expressed activity (100% expressed activity equals initial activity 9.89 µmol/min mL).

Not surprisingly, by increasing the concentration of cellulase from 25.0 mg/mL to 37.5 mg/mL in CLEAs and mCLEAs, their activity decrease was observed (Figure 5). It was reported that the enzyme concentration influenced the amount and particle size of CLEAs [78]. Every enzyme has a unique structure, and the optimum activity was obtained at a certain shape and size of particles. Increasing enzyme concentration can lead to diffusional limitations and the loss of activity, when enzyme aggregates are packed together [79]. Excessive protein may result in compact aggregates, which can lead to a loss of flexibility. It can also hinder active sites of the enzyme from attaching to the substrate. Similar results were also observed in previous studies [80]. Immobilization yield was 100% for both CLEAs and mCLEAs with 0.125% (*w*/*w*) GA at 25 °C. When the concentration of the enzyme in CLEAs and mCLEAs was 25 mg/mL, the expressed activity was 112.14% (11.09 µmol/min mL) for CLEAs and 148.29% (14.67 µmol/min mL) for mCLEAs. With the increase in enzyme concentration, the expressed activity decreased below 100% and was 84.22% (8.33 µmol/min mL) for CLEAs and 97.10% (9.60 µmol/min mL) for mCLEAs. It can be assumed that the enzyme immobilization already reached its maximum and that the surface of the nano-carrier became saturated with the enzyme. The aggregation of the enzyme, which makes some active sites of the enzyme hidden, may also be the cause for a decrease in activity at higher enzyme concentrations. The same effect was already confirmed with our previous studies [75] and also observed by Eldin et al., where higher enzyme concentrations caused the increase in density of the immobilized enzyme molecules on the particles’ surface. Such an occurrence later led to higher protein-to-protein interactions, which caused the reduction in catalytic activity [81]. Immobilization yield was 100% for both CLEAs and mCLEAs with 0.125% (*w*/*w*) GA, at 25 °C. When the concentration of the enzyme in CLEAs and mCLEAs was 25 mg/mL, the expressed activity was 134.57% (11.09 µmol/min mL) for CLEAs and 148.29% (14.67 µmol/min mL) for mCLEAs. With the increase in enzyme concentration, the expressed activity decreased and was 84.22% (8.33 µmol/min mL) for CLEAs and 97.10% (9.60 µmol/min mL) for mCLEAs.

### 2.4. Effect of Increasing GA Concentration

**Figure 6:**
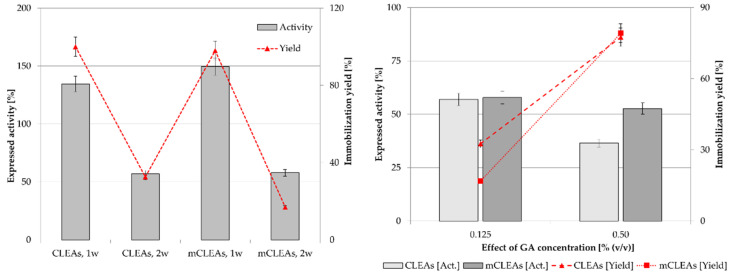
Influence of the two-time washing cycle (1-propanol, 0.125% (*w*/*w*) GA, 1.88 mg/mL BSA at 25 °C) on CLEAs and mCLEAs expressed activity and immobilization yield (100% expressed activity equals initial activity 9.89 µmol/min mL).

In the next phase of the CLEAs and mCLEAs preparation procedure, the CLEAs and mCLEAs were finally recovered by centrifugation and purified by washing two times. Uncross-linked enzymes were removed and assayed in supernatant. Based on previous results of this study, CLEAs and mCLEAs were prepared with 1-propanol as the precipitating reagent in the amount of 0.125% (*w*/*w*) GA at the temperature of cross-linking at 25 °C. The concentration of BSA was 1.88 mg/mL and the enzyme concentration was 25 mg/mL. Activity drop after washing was also observed in a report by Galvis et al., which can be attributed to the larger sizes of the particles which form after centrifugation, where intense inter-particle cross-linking is happening, which can distort the enzyme and therefore increase diffusional limitations [77]. The results with this optimized synthesis gave expressed activity of 112.14% (11.09 µmol/min mL) for CLEAs and 148.29 (14.67 µmol/min mL) for mCLEAs. The immobilization yield was 100 % for CLEAs and 98.02% for mCLEAs. Figure 6 shows a considerable drop in activity and yield after washing. This activity and immobilization yield reduction after the washing procedure could be explained by the insufficient degree of cross-linking. When dextran [82] in a concentration of 0.25 % (*w*/*w*) was used as a cross-linker instead of GA, the activity decreased by 57% suggesting that in the given conditions of cross-linking better results were achieved using GA as a cross-linker. One possibility to improve the stability of CLEAs after washing was to further increase the GA concentration to 0.50% (*w*/*w*).

**Figure 7:**
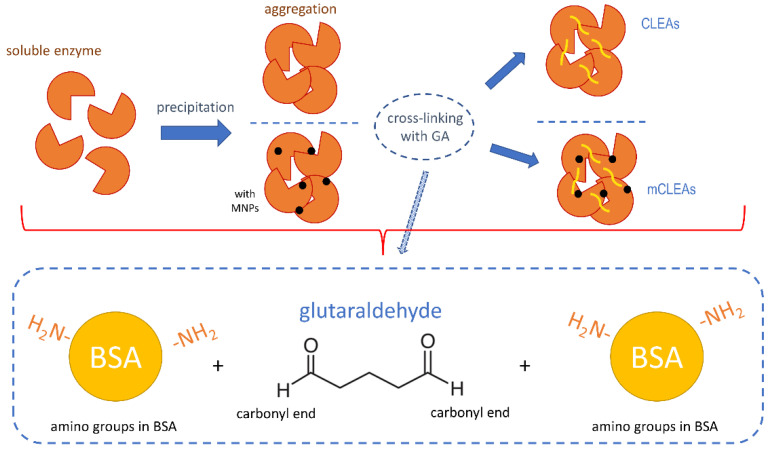
Schematic mechanism of cross-linking with GA in CLEAs and mCLEAs synthesis.

GA is in reaction with proteins involved in the formation of Schiff bases between two carbonyl ends of GA and positively charged amino groups on the surface of certain proteins (schematic presented in Figure 7). Additionally, monomeric GA can easily be polymerized by condensation, which enhances the mixtures of elongated species. This can also cross-link intramolecular and intermolecular lysines in a non-specific manner [83]. In the case of low GA concentration, cross-linking is incomplete and the stability of CLEAs in aqueous media is too low [84]. However, too high a concentration of GA may cause a significant loss of enzyme activity [66].

Increasing GA concentration resulted in a considerable increase in the yield (for 35% and more) and a decrease in CLEAs and mCLEAs activity, suggesting enzyme damage due to excessive cross-linking (Figure 6). Therefore, excessive GA concentrations can cause a decrease in enzyme activity, since GA acts as a protein denaturant as well. This can cause significant changes in the protein structure. Moreover, the flexibility of the enzyme becomes limited, and the rigidity of the enzyme prevents its substrate from reaching the active sites, thus increasing the steric hindrance. In order to achieve an active immobilized enzyme, optimum cross-linker concentrations must be applied to the synthesis process [85,86,87].

### 2.5. Effect of Reducing Agent Concentration

**Figure 8:**
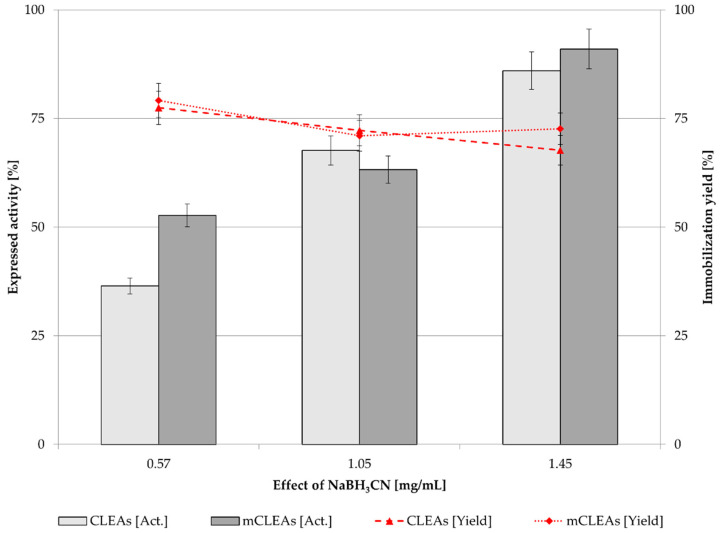
Effect of NaBH_3_CN (0.1 M) concentration on CLEAs and mCLEAs expressed activity and binding yield (0.50% (*w*/*w*) GA, two-time washing, at 25 °C) (100% expressed activity equals initial activity 9.89 µmol/min mL).

NaBH_3_CN is used as a reducing agent in the cross-linking synthesis that can convert imines to amines. It reduces the unsaturated Schiff’s base to form irreversible linkages [88]. Increasing the concentration of NaBH_3_CN in CLEAs and mCLEAs to 1.45 mg/mL significantly increased their activity. For CLEAs, the expressed activity increased by 50.08% (from 35.49% to 85.57%), and for mCLEAs, the expressed activity increased by 38.56% (from 53.79% to 92.35%), as can be seen in Figure 8. It is assumed that there are approx. 12% more unsaturated linkages of Schiff’s bases in CLEAs than in mCLEAs, where the amino functionalized MNPs decreased the number of unsaturated linkages.

NaBH_3_CN reduced the unsaturated Schiff’s bases to form irreversible linkages [66,82]. The advantage of using a slightly weaker reducing agent, such as sodium cyanoborohydride, is that it can be added to the reaction mixture to only reduce the specific iminium salt. As it is not a strong enough reducing agent, it will only reduce iminium ions. In another study, the stability of CLEAs and mCLEAs in aqueous media was improved [4]. The presence of stronger inter- and intra-molecular cross-linking as well as related active conformation of the enzyme were evident, leading to higher activity and stability. No significant change in immobilization yield was observed. A study by Khorshidi et al. reports on cellulase mCLEAs which were prepared with improved thermal stability at 65 °C. Optimum pH and temperature were determined. Cellulase retained about 40% of its original activity after immobilization in the form of mCLEAs [89]. Another study describes preparation of cross-linked enzyme aggregates from cellulase with more emphasis on structural characterization and analysis, investigating the pH and temperature of immobilized cellulase [87], while our study investigates the effect of proteic feeder on enzyme activity and stability, as well as the effect of GA and a reducing agent on cellulase activity while using MNPs in magnetic cross-linked enzyme aggregates. The activity of mCLEAs is 7% higher (92.35%) than of CLEAs (85.57%), where there are no MNPs implemented in the immobilization system.

### 2.6. Reusability of CLEAs and mCLEAs

**Figure 9:**
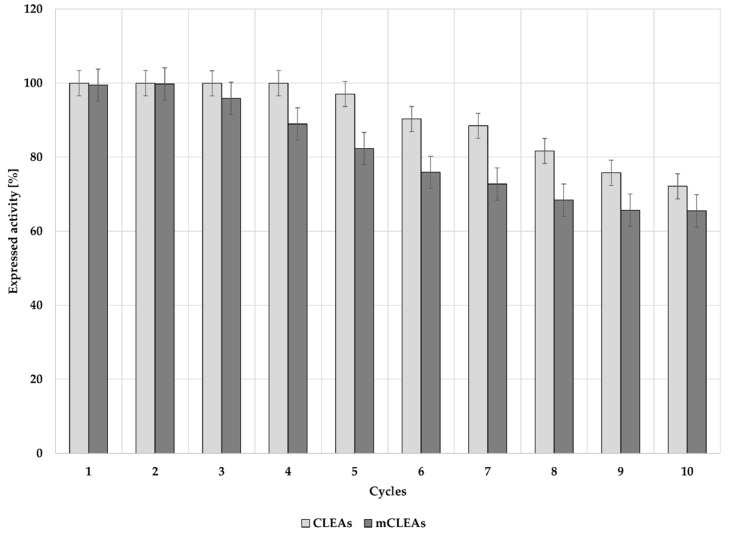
Recyclability of CLEAs and mCLEAs after 10 cycles.

The choice between free soluble enzyme and enzyme in insoluble form in industry is largely determined by the cost of the enzyme and its intended use in certain applications. Insoluble enzymes are mostly in immobilized form, where they are specialized for the use in heterogeneous catalysis with the ability to be recovered and further reused for more operational cycles, which is an indirect feature of its total productivity. However, the cost of immobilized enzymes is also determined on their reaction kinetics and specificity [90,91]. Reusability of CLEAs and mCLEAs was studied and the results are presented in Figure 9. Both CLEAs and mCLEAs retained 100% of initial activity after 4 cycles for CLEAs and after 2 cycles for mCLEAs. Their activity slightly decreased with each further cycle of reuse and throughout 10 cycles of reuse, CLEAs and mCLEAs did not reach half-life, but retained 72% and 65% of their initial activity, respectively. Additionally, GA increased the size of the enzyme clusters and gave a slight shift in the pH activity curve, which can also increase the cellulase stability and activity. These results suggest that cross-linking provides a higher stability and reduces enzyme leaching during the reuse of the immobilized cellulase. Previous studies show cellulase immobilization as mCLEAs to retain 74% of the original activity after 6 cycles [87] and 55% after 4 cycles when immobilized on different magnetic supports [92].

### 2.7. Thermal Stability of CLEAs and mCLEAs

**Figure 10:**
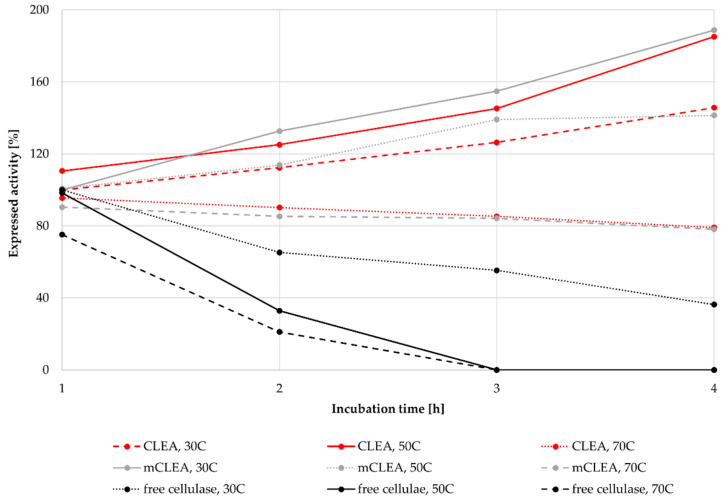
Thermal stability of CLEAs, mCLEAs, and free cellulase at 30 °C, 50 °C, and 70 °C, incubated for 4 h.

As many industrial processes run at elevated temperatures for longer periods of time, such processes require thermally stable enzymes. Many industrial enzymes must withstand higher temperatures during different bioprocesses. For cellulosic bioprocesses, used enzymes must endure temperatures usually around 50 °C for up to 72 h. The stability of CLEAs and mCLEAs at 30 °C, 50 °C, and 70 °C was determined. CLEAs and mCLEAs were prepared with an enzyme concentration of 25 mg/mL, using 1-propanol as the precipitating reagent and a cross-linking time of 3 h. The concentration of GA was 0.125% (*w*/*w*) and the concentration of NaBH_3_CN was 1.45 mg/mL. The concentration of BSA was 1.88 mg/mL for CLEA and 1.25 mg/mL for mCLEA. Both CLEAs and mCLEAs were incubated at each temperature for 4 h in order to determine their stability. The activity of CLEAs and mCLEAs increased significantly in comparison with the activity of free enzyme after 4 h of incubation at each temperature. When incubating CLEAs at 30 °C for 4 h, their activity increased by 45.65%, resulting in 145.65% of its initial activity. Similar results were obtained after incubation of mCLEAs at 30 °C, where after 4 h the activity of mCLEAs increased for 88.7%. The increase in the immobilized cellulase activity is probably attributed to the cross-linking of enzyme molecules that kept the conformational integrity and thus the catalytic activity [93] as can be seen in Figure 10. When incubating CLEAs and mCLEAs at 50 °C, their activity still increased after 4 h, resulting in 185.1% and 141.4%, respectively. An additional thermal stability test was performed at 70 °C, where the activity of CLEAs and mCLEAs decreased slightly, but still retained 79.1% and 78.1%, respectively. For comparison, the thermal stability of free cellulase was investigated as well. Free cellulase retained only 36.3% of its initial activity after 4 h, whereas at incubation at 50 °C and 70 °C the free cellulase was inactive after 4 h of incubation. The results suggest that immobilized CLEAs and mCLEAs of cellulase show good thermal stability compared with free cellulase at specific temperatures. Our results are consistent with other studies, where the temperature for the highest activity of free cellulase was at 50 °C and shifted to 60 °C for mCLEAs [87]. Such results may be a result of immobilization scaffold occurring, which can prevent the stretching of enzyme molecules at elevated temperatures.

For a more reliable comparison of cellulase CLEAs and mCLEAs, the preparation was in all phases parallel and under the same conditions. The immobilization yield was 100% for CLEAs and 99% for mCLEAs. The impact of additives (BSA) and influence of enzyme concentration was tested. With the addition of NaBH_3_CN, the linkages in CLEAs and mCLEAs were again stabilized, which was proven by the stability of CLEAs and mCLEAs at all tested temperatures in comparison with the free enzyme, which became inactive after 4 h of incubation.

### 2.8. Determination of Kinetic Parameters

To determine the *K*_M_ and *V*_max_ of free cellulase, CLEAs, and mCLEAs, the peak area of the product for a series of concentrations of Sigmacell cellulose substrate was determined. The kinetic parameters (*K*_M_ and *V*_max_) of free cellulase, CLEAs, and mCLEAs are shown in Table 2, where the *K*_M_ values of free cellulase, CLEAs, and mCLEAs were 0.012 ± 0.0018 mM, 0.055 ± 0.0102 mM, and 0.037 ± 0.0012 mM, respectively. Compared with the free cellulase, the CLEAs and mCLEAs have a higher *K*_M_, which indicates that the immobilized cellulase in the form of CLEAs and mCLEAs has a lower affinity towards substrate cellulose. This may be because the structure of the cellulase becomes more compact after cross-linking, thus the accessibility of the substrate to the cross-linked enzymes’ active sites is restricted [59,94]. The *V*_max_ of CLEAs and mCLEAs (1.12 ± 0.0012 and 1.17 ± 0.0023 µmol/min, respectively) decreased compared with free cellulase (8.86 ± 0.0041 µmol/min), which can be attributed to some steric hindrance of the enzyme to their polymeric substrates. Similar observations were made by Shuddhodana [95] and Bhushan [96].

### 2.9. XRD Analysis

**Figure 11:**
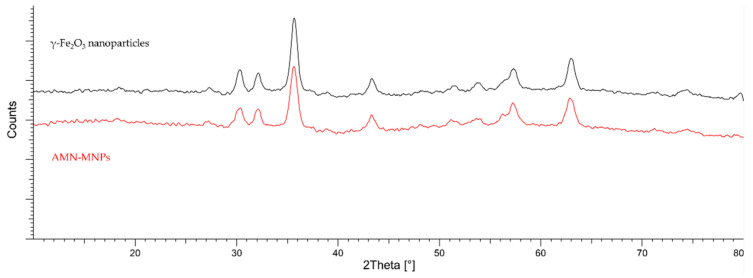
Simulated XRD patterns simulated of maghemite (γFe_2_O_3_) and synthesized AMN-MNPs.

Surface characterization of synthesized AMN-MNPs was published in our previous research by Leitgeb et al. and can be found in [97]. The XRD spectra provide information about the crystalline structure of synthesized MNPs, as well as their degree of structural order. Figure 11 shows the XRD patterns of coated AMN-MNPs and bare γ-Fe_2_O_3_ nanoparticles. Diffraction peaks obtained for AMN-MNPs and bare γ-Fe_2_O_3_ nanoparticles are in correlation with the simulated diffractogram for maghemite, displaying the formation of the iron oxide phase, which shows that further surface functionalization with aminosilane maintained its crystalline structure. The values of the particles’ sizes, calculated using Scherrer’s equation, are given in Table 3.

### 2.10. SEM Analysis

**Figure 12:**
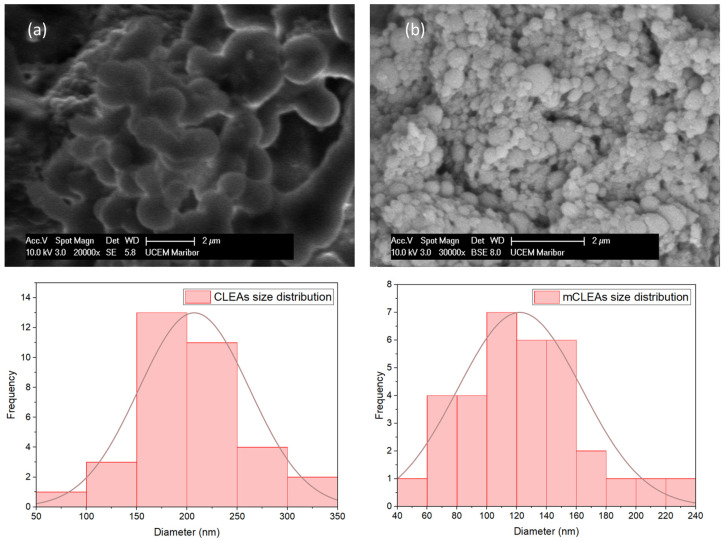
SEM images of (**a**) CLEAs and (**b**) mCLEAs with their corresponding particle size distributions.

The surface morphology of prepared CLEAs and mCLEAs was analyzed by scanning electron microscope (SEM). The synthesis of CLEAs and mCLEAs was confirmed by representative SEM images, as well as their corresponding particle-size distributions, which are shown in Figure 12. Figure 12a shows CLEAs prepared in 1-propanol, where spherical aggregates were observed, with their corresponding particle size distribution that resulted in an average diameter of 180 nm. Figure 12b shows mCLEAs, also prepared in 1-propanol, which displayed spherical aggregates as well and the particle size distribution resulted in an average diameter of 110 nm.

### 2.11. FT-IR Analysis

**Figure 13:**
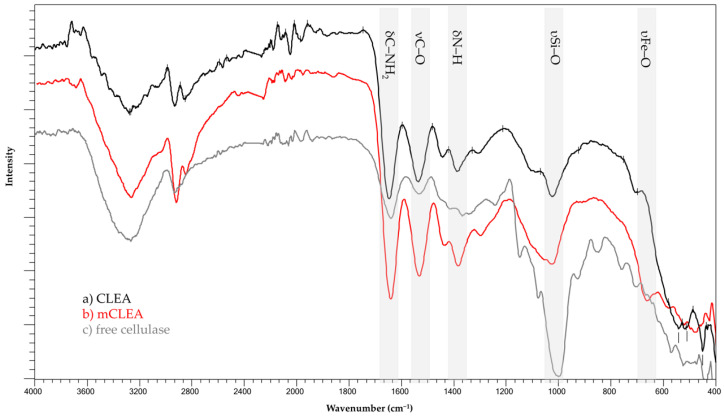
FT-IR spectra of CLEAs, mCLEAs, and free cellulase.

Figure 13 shows the FT-IR spectra of the samples of CLEAs, mCLEAs, and free cellulase. In the mCLEAs FT-IR spectra, the bands assigned to the υFe–O stretching mode appear around 650 cm^−1^. AMN-MNPs that are present in mCLEAs are confirmed in the FT-IR spectrum with the band centered at 1010 cm^−1^ assigned to υSi–O bond stretching mode [74]. In addition, the band in the region of 1640–1540 cm^−1^ is attributed to the bending modes from the δC–NH_2_ bond [31]. Lastly, the immobilization of cellulase in the form of CLEAs and mCLEAs surfaces is observed with bands centered around 1530 cm^−1^ and 1400 cm^−1^, and assigned to νC–O bond stretching mode and δN–H bending mode, respectively. All peaks indicate that the cellulase is successfully attached to the support and all obtained peaks are well matched with earlier supports [98,99,100].

## 3. Materials and Methods

### 3.1. Materials

The cellulase Cellusoft (EC 3.2.1.4) was kindly obtained from Novozymes A/S (Denmark), GA solution (25%), bovine serum albumin (BSA), CMC (carboxymethylcellulose), DNS (3,5-dinitrosalicylic acid, citrate buffer, and glucose standard were supplied by Sigma-Aldrich, Darmstadt, Germany). Ammonia solution (25%) was supplied by Chem-Lab (Belgium). Sodium cyanoborohydride (NaBH_3_CN), trichloromethane, 1-propanol, acetonitryle, sodium dihydrogen phosphate (NaH_2_PO_4_), Coomassie brilliant blue, ortho-phosphoric acid (88% (*v*/*v*)), iron (II), chloride-4-hydrate (FeCl_2_ × 4H_2_O), iron (III) chloride-6-hydrate (FeCl_3_ × 6H_2_O), citric acid, and sodium chloride were purchased from Merck (Darmstadt, Germany). Methanol and acetone were supplied by Carlo Erba (Carnaredo, Italy). Ethanol was obtained from Kefo (Slovenia), glycerol was purchased from Kemika (Zagreb, Croatia), and 2-propanol, sodium silicate (Na_2_SiO_3_), and 3-aminopropyl-trimethoxysilan (APTMS) (97%) were obtained from Sigma-Aldrich (Darmstadt, Germany).

### 3.2. Preparation of Aminosilanized Magnetic Nanoparticles (AMN-MNPs)

The maghemite nanoparticles were synthesized by co-precipitation of Fe^2+^ and Fe^3+^ ions in a molar ratio of 2:1 with a concentrated ammonium solution (25%) at room temperature by the method described in our previous work [101]. Firstly, the pH of the solution was adjusted dropwise with ammonium solution to pH 3 and stirred. In this stage, Fe(OH)_3_ was precipitated. Secondly, concentrated ammonium solution was added to the mixture, where pH was raised to 11 and stirred, which gave the resulting precipitate of maghemite. The precipitant was separated by magnetic decantation.

Magnetic fluid was prepared to coat the synthesized maghemite nanoparticles using citric acid. To the precipitant, distilled water and citric acid (33%) was added, which was later on stirred for another 90 min at 75 °C. After cooling, the pH was adjusted to 10 with ammonium solution and centrifuged for 5 min at 5000 rpm. Magnetic fluid was prepared for silanization.

In the first step of silanization, the magnetic nanoparticles were coated with silica. To 60 mL of magnetic fluid, silica (Na_2_SiO_3_) solution was added at 85 °C to 90 °C by stirring for 3 h. Coated nanoparticles were centrifuged and dried. In the second step, nanoparticles were silanized by the addition of aminosilane (APTMS) with pH adjusted to 4. To the dried silica-coated nanoparticles, methanol and glycerol were added. The suspension was heated by stirring at 90 °C and aminosilane (APTMS) solution was added dropwise. The mixture was centrifuged. The resulting amino-functionalized magnetic nanoparticles were washed with NaCl solution and finally centrifuged and dried [97].

### 3.3. Preparation of CLEAs and mCLEAs

CLEAs and mCLEAs of cellulase were prepared in three steps: preparation of enzyme solution (CLEAs and mCLEAs), precipitation or physical aggregation, and cross-linking. All phases were performed at room temperature (T = 25 °C).

#### 3.3.1. Preparation of Enzyme Solution for CLEA

The enzyme solution was prepared with cellulase (Cellusoft) in a concentration of 250 mg/mL, phosphate buffer (PBS; 0.02 M, pH 7), and additives; if necessary, bovine serum albumin (BSA; 5%) was added taking into consideration that the final volume of enzyme solution was always 400 µL. The prepared enzyme solution was stirred for 40 min.

#### 3.3.2. Preparation of Enzyme Solution for mCLEA

When preparing mCLEAs, aminosilanized MNPs were added to the enzyme solution as described in Section 3.3.1. 

#### 3.3.3. Enzyme Precipitation

Enzyme solution was added dropwise at room temperature into different precipitation reagents (acetone, ethanol, 1-propanol, and 2-propanol) in a volume ratio of 1:9, where all precipitation reagents exhibited neutral pH (pH 7). The mixture was stirred for another 40 min and later centrifuged at 11,000 rpm for 2 min. The protein concentration and enzyme activity in supernatants were determined, as well as the enzyme activity, on obtained pellets. Based on the results, the most suitable organic solvents as precipitation reagents were selected.

#### 3.3.4. Cross-Linking of CLEAs and mCLEAs

An amount of 100 µL of previously prepared enzyme solution was added dropwise to 900 µL of precipitation reagent (selected organic solvents) [102]. The mixture (25.0 mg/mL of cellulase, 1.25 mg/mL of MNPs, and precipitant) was stirred for 40 min. Later on, GA was added to the mixture with three different final concentrations (0.50% (*w*/*w*), 0.125% (*w*/*w*), and 0.05% (*w*/*w*)). The mixture was stirred for an additional 3 h. NaBH_3_CN (100 mM) was added in different concentrations (0.57 mg/mL, 1.05 mg/mL, and 1.45 mg/mL) to form irreversible linkages and the suspension was stirred for a further 40 min. Prepared CLEAs were separated by centrifugation for 2 min at 11,000 rpm, and mCLEAs were separated with magnetic decantation. The protein concentration in the supernatant and the enzyme activity on the pellets was measured. The first phase (optimization of concentrations of cross-linker, BSA, precipitant, and concentration of enzyme) of CLEAs and mCLEAs preparation was finalized with centrifugation, whereas the centrifugation in the second phase was followed by CLEAs and mCLEAs purification by two washings with 1 mL of buffer PBS (0.02 M, pH 7).

### 3.4. Determination of Immobilization Yield

The protein concentration in the supernatant of all samples measured using the Bradford spectrophotometric method [103] was 595 nm, using BSA as the standard for the calibration curve. The immobilization yield was calculated by the following Equation (1):

(1) $$\mathrm{Immobilization}\;\mathrm{yield}\;(\%)\;=\;\frac{\left(c_{i}-c_{s}\right)}{c_{i}}\times 100$$

where:

c*_i_* = concentration of cellulase initially used for reaction.
c*_s_* = concentration of unbound cellulase, collected in the supernatant and in each purification (washing) cycle, respectively.

### 3.5. Cellulase Activity Assay

The cellulase activity (U/mL) of both immobilized and free cellulase was determined using CMC as the substrate, 2% (*w*/*v*) CMC in 50 mM citrate buffer (pH 4.8), where the reducing end concentration was measured by the DNS method. The activity towards CMC was measured as reducing sugars formed and is understood as endoglucanase activity [104]. Immobilized and free cellulose was equilibrated to 50 °C, and 0.5 mL of CMC solution was added and incubated for 30 min at 50 °C. After 30 min, 3 mL of DNS solution was added and mixed. Prepared samples were boiled for 5 min and later placed on ice bath to quench the reaction. An amount of 20 mL of distilled water was added and the absorbance was measured at 540 nm. The substrate blank contained 0.5 mL of CMC solution and 0.5 mL of citrate buffer. Glucose released by the enzyme solution was calculated from the glucose standard curve. The expressed activity (%) was expressed as the ratio between the measured activity of the CLEAs (U/mL) and the measured activity of the free enzyme (U/mL, standard solution), following Equation (2):

(2) $$\mathrm{Expressed}\;\mathrm{activity}\;\left(\%\right)=\frac{\left(\mathrm{activity}\;\mathrm{of}\;\mathrm{CLEAs}\;\mathrm{or}\;\mathrm{mCLEAs}\right)}{\mathrm{activity}\;\mathrm{of}\;\mathrm{free}\;\mathrm{enzyme}}\times 100$$

where:

activity of CLEAs or mCLEAs = activity of cross-linked cellulase or magnetic cross-linked cellulase measured by cellulase activity assay (U/mL).
activity of free enzyme = activity of free cellulase measured by cellulase activity assay (U/mL).

### 3.6. Reusability Studies of Free Cellulase, CLEAs, and mCLEAs

The reusability of immobilized CLEAs and mCLEAs were assessed. After each cycle, the immobilized CLEAs and mCLEAs were separated by centrifugation for 2 min at 11,000 rpm and 4 °C and washed with sodium acetate buffer (50 mM and pH 5.0) for the next cycle reuse. In running the second cycle, the immobilized enzyme was redissolved in fresh buffer and the mixture for determining enzyme activity was added to the sample. Later, activity was assessed in the same way as before. Initial activity of the immobilized enzyme was considered as 100%.

### 3.7. Thermal Stability of Free Cellulase, CLEAs, and mCLEAs

Stability of the free cellulase and of the immobilized enzyme CLEAs and mLCEAs was determined at 30 °C, 50 °C, and 70 °C. CLEAs and mCLEAs were prepared according to the method described before with optimal parameters and incubated at 30 °C, 50 °C, and 70 °C for 4 h. After each hour, respective activity changes were determined. The activity after incubation at different temperatures was expressed as the expressed activity (%) assuming that the initial activity of CLEAs and mCLEAs after the preparation was 100%.

### 3.8. Determination of Kinetic Parameters

The enzyme kinetic parameters, Michaelis–Menten constant (*K*_M_), and maximum reaction velocity (*v*_max_) are characteristic kinetic constants that are used to evaluate the performance of immobilized enzymes. *K*_M_ can be calculated using the Lineweaver–Burk diagram under optimum conditions. The Michaelis constants (*K*_M_) and the maximum reaction rate (*v*_max_) of free cellulase, CLEAs, and mCLEAs were determined using different concentrations of the Sigmacell cellulose substrate (2.5%, 5%, 7.5% and 10%, *w*/*v*) in sodium acetate buffer (50 mM and pH 5.0) at 37 °C. Non-linear regression analysis was applied to calculate the value of *K*_M_ and *V*_max_.

### 3.9. X-ray Diffraction Analysis

X-ray diffraction powder analysis was carried out with a Bruker D2 Phaser diffractometer (Cu–Kλ radiation; 1.5406 Å), covering the range of diffracting angles 2Θ from 10° to 80°, measuring with a 0.03° step and a time/step of 1 s. Dried powder samples were dispersed in isopropanol, deposited on a sample holder, and dried in order to obtain a thin layer of particles. Recorded XRD scattering patterns were used for calculation of the average crystal size of the synthesized maghemite and coated nanoparticles, implementing Scherrer’s equation from a reflection peak of (3 1 1).

### 3.10. Scanning Electron Microscopy Analysis

SEM analysis was performed using a scanning electron microscope (FE, SEM SIRION, 400 NC, and FEI) to investigate the morphology and size of the prepared AMN-MNPS, cellulase CLEAs, and cellulase mCLEAs. The samples were measured on a gold (Au) substrate. Moreover, energy-dispersive X-ray spectroscopy (EDX) microanalysis was performed for chemical composition analysis.

### 3.11. Fourier Transform Infrared Spectroscopy (FT-IR)

To study chemical bonds formed between AMN-MNPS, cellulase CLEAs, and cellulase mCLEAs, FT-IR analysis of the samples was performed by pressing the samples to form a tablet using KBr as the matrix. The spectra were detected over a range of 4000–500 cm^−1^ and recorded by a FT-IR spectrophotometer (Perkin Elmer 1600 Fourier transform infrared spectroscopy).

## 4. Conclusions

Since cellulases are enzymes which catalyze the hydrolysis of cellulose into oligosaccharides and glucose, its utilization into various industrial processes, such pulp and paper treatment, biofuel production, textile, and food processing, is of high importance. Different approaches have been investigated to immobilize cellulases in order to achieve reusability and improved thermal stability, as well as to improve their hydrolytic activity. Immobilization of cellulase via precipitation and cross-linking is the most cost-effective and simple method to obtain reusable biocatalysts. To achieve such reusable, thermostable biocatalysts with improved activity, cellulase CLEAs and mCLEAs were parallelly synthesized. Immobilization through CLEAs and mCLEAs synthesis involves the entire surface of the enzyme in the immobilization and presents some advantages in comparison with more traditional immobilization methods, such as easy and simple preparation [14,20]. It also exhibits high catalyst productivities, which were confirmed in our studies with improved expressed activities through careful optimization of parameters. Reaction conditions were optimized to improve the activity of CLEAs and mCLEAs. The expressed activity of resulted CLEAs was 85.57% (8.46 µmol/min mL) and for mCLEAs 92.35% (9.13 µmol/min mL). The addition of a reducent NaBH_3_CN improved the expressed activity of CLEAs for 50% and mCLEAs for 38.56% due to reducing the unsaturated Schiff’s base to form irreversible linkages. When CLEAs and mCLEAs were incubated at 30 °C and 50 °C, higher activities of both were observed in comparison with the activity of non-immobilized enzymes. Expressed activities for CLEAs and mCLEAs increased by approx. 45% and 88% after 4 h of incubation at 30 and 50 °C in comparison with the initial activity, while non-immobilized cellulase at the same conditions was inactivated. The results of this study show that the addition of amino-functionalized MNPs improves the expressed activity of cellulase-mCLEAs, as the expressed activity is slightly higher than CLEAs cellulase. Reusability after 10 cycles was investigated for both CLEAs and mCLEAS, which retained 72% and 65% activity, respectively. Kinetic parameters were determined and calculated, as well as compared with free cellulase. The *K*_M_ constant was found at 0.055 ± 0.0102 mM and 0.037 ± 0.0012 mM, respectively. The maximum velocity rate (*V*_max_) was calculated as 1.12 ± 0.0012 µmol/min for CLEA and 1.17 ± 0.0023 µmol/min for mCLEA. Structural characterization was studied using XRD, SEM, and FT-IR, which confirmed successful enzyme aggregation and synthesis of CLEAs and mCLEAs using AMN-MNPs. Hyperactivation of the enzymes occurred, which can happen when free enzyme is highly unstable, and the immobilization improves enzyme stability and catalytic performance. The phenomenon of hyperactivation most likely occurs due to the conformational changes in the protein in the form of clusters or aggregates [64]. It can be seen that the addition of functionalized aminosilane MNPs has many benefits for enzyme immobilization because it improves catalytical and non-catalytical properties of the enzyme. The addition of MNPs has been proved to enhance the activity of mCLEAs. Additionally, after the reaction, mCLEAs can be separated from the reaction mixture by a simple magnetic decantation. Further, MNPs provide additional mechanical stability of cross-linked enzyme aggregates. Therefore, mCLEAs have many applications as well, since MNPs are useful to simplify the handling of products, even during the preparation process. Such mCLEAs have many applications in different areas, especially in hyperthermia [36]. The use of such (magnetic) biocatalysts in bioprocess technology leads to an efficient separation and handling, as well as reutilization of the biocatalyst. However, there are still some scientific and technical challenges that must be addressed in order to achieve more economically feasible applications in optimizing the immobilization protocol.

## Figures and Tables

**Table 1:** Relative activity (%) and immobilization yield (%) for CLEAs and mCLEAs using 1-propanol as precipitating agent.

	Activity CLEAs	Activity mCLEAs	Yield CLEAs	Yield mCLEAs
0.50% (*w*/*w*) GA	181.71	174.79	99.76	99.69
0.125% (*w*/*w*) GA	152.34	161.33	99.96	99.89
0.05% (*w*/*w*) GA	87.15	124.63	100	100

**Table 2:** Kinetic analysis of free cellulase, CLEAs, and mCLEAs on the hydrolysis of Sigmacell cellulose.

Sample	*V*_max_ [µmol/min]	*K*_M_ [mM]
Free cellulase	8.86 ± 0.0041	0.012 ± 0.0018
CLEAs	1.12 ± 0.0012	0.055 ± 0.0102
mCLEAs	1.71 ± 0.0023	0.037 ± 0.0012

**Table 3:** The values of synthesized MNPs, determined with Scherrer’s equation.

Sample	d_XRD_ [nm]
γ-Fe_2_O_3_	11.2
AMN-MNPS	12.4

## Data Availability

All data generated or analyzed during this study are included in this published article.

## Sample Availability

Samples of the compounds are available from the authors.
